# Why Are Workplace Social Support Programs Not Improving the Mental Health of Canadian Correctional Officers? An Examination of the Theoretical Concepts Underpinning Support

**DOI:** 10.3390/ijerph18052665

**Published:** 2021-03-06

**Authors:** Geneviève Jessiman-Perreault, Peter M. Smith, Monique A. M. Gignac

**Affiliations:** 1 Dalla Lana School of Public Health, University of Toronto, Toronto, ON M5T 3M7, Canada; psmith@iwh.on.ca (P.M.S.); mgignac@iwh.on.ca (M.A.M.G.); 2 Institute for Work and Health, Toronto, ON M5G 1S5, Canada

**Keywords:** interpersonal support, workplace health, mental health, corrections, public safety personnel

## Abstract

In Canada, public safety personnel, including correctional officers, experience high rates of mental health problems. Correctional officers’ occupational stress has been characterized as insidious and chronic due to multiple and unpredictable occupational risk factors such as violence, unsupportive colleagues and management, poor prison conditions, and shift work. Given the increased risk of adverse mental health outcomes associated with operational stressors, organizational programs have been developed to provide correctional officers with support to promote mental well-being and to provide mental health interventions that incorporate recovery and reduction in relapse risk. This paper uses two theories, the Job Demand Control Support (JDCS) Model and Social Ecological Model (SEM), to explore why workplace social support programs may not been successful in terms of uptake or effectiveness among correctional officers in Canada. We suggest that structural policy changes implemented in the past 15 years have had unintentional impacts on working conditions that increase correctional officer workload and decrease tangible resources to deal with an increasingly complex prison population. Notably, we believe interpersonal support programs may only have limited success if implemented without addressing the multilevel factors creating conditions of job strain.

## 1. Introduction

In Canada, public safety personnel (PSP) (i.e., front-line personnel whose job roles include ensuring the safety and security of Canadians) [1] experience high rates of mental health problems such as depression, anxiety [2], post-traumatic stress disorder (PTSD) [3], suicidal ideations [4], and alcohol use disorders [5]. Among 6 categories of Canadian PSP (emergency dispatchers, correctional workers, firefighters, municipal/provincial police, paramedics, and Royal Canadian Mounted Police (RCMP)), 26.7% reported mental health symptoms consistent with more than one mental disorder [2]. These elevated risks have been attributed to the operational stressors inherent to the work of PSP, including harassment, violence, and risk of death [6].

Some provincial and territorial governments in Canada have recognised the mental health risks experienced by PSP by categorizing many of these professionals as presumptively covered for PTSD claims under provincial or territorial worker’s compensation programs [7]. While these policy changes have been a positive movement towards recognising the mental health burden of high-stress and high-risk work, preventive strategies are also needed to reduce the human, social, and economic costs of adverse mental health outcomes in this population.

Among PSP there exists a diversity of occupational roles, responsibilities, and risks. This paper focuses on correctional officers in Canada who are necessary first responders and occupy many roles within correctional institutions [8]. The correctional workplace requires officers to provide care, custody, and control for those housed within correctional institutions, which can include providing emergency medical care, firefighting services, and verbal or physical de-escalation [9]. Among PSP, correctional officers experience a high burden of mental health outcomes, with one study finding that 54.6% of correctional officers screened positive for one or more mental health disorders compared to 44.5% of PSP overall [2]. Correctional officers’ occupational stress has been characterized as insidious and chronic due to the multiple and unpredictable occupational risk factors they face such as violence, unsupportive colleagues and management, poor prison conditions, and shift work [10].

Given the range of operational stressors and their link to an increased risk of adverse mental health outcomes, organizational programs have been developed to provide correctional officers with supports aimed at promoting mental well-being and providing mental health interventions that incorporate recovery and reduction of relapse risk [11,12,13]. However, research has shown minimal support for the effectiveness of such individualistic workplace mental health programs [13,14]. A recent international scoping review that examined prevention and early intervention programs for veterans and first responders (including correctional officers) found that the mental health interventions that have been implemented typically focus on building individual-level resilience and interpersonal support programs [13]. The authors also determined that there are no validated mental health interventions being implemented in this population and workplaces typically do not evaluate their programing using accepted methods, therefore, evidence for effectiveness of individual and interpersonal mental health programming is lacking [13]. This finding is supported by a recent systematic review focused on peer support and crisis-focused interventions implemented among PSP and healthcare personnel [14]. The authors found that the majority of the studies included in their review did not conduct pre-post evaluations and used inconsistent outcome measures which resulted in poor quality of evidence of the effectiveness of such interventions [14].

Despite these methodological challenges, recent research has identified that social support has the potential to reduce the burden of PTSD and major depressive disorders (MDD) among PSP. Vig et al. [15] found that PSP with higher self-reported perceived social support were 7–10% less likely to screen positively for PTSD and 11–15% less likely to screen positively for MDD. Yet, uptake of interpersonal support programming among correctional officers has been shown to be low [16]. Taken together, these findings could indicate that while social support has the potential to reduce adverse mental health outcomes among PSP, interpersonal workplace support may not be accepted as a viable source of support among correctional officers. 

To further understand the findings evaluating support programs and to highlight new directions for the development, implementation and evaluation of support programs, this paper draws on two theories, the Job Demand Control Support (JDCS) Model and Social Ecological Model (SEM). Elsewhere, much has been written about the limitations of workplace mental health interventions that focus solely on individual behaviour change (e.g., resilience-building programs) in the absence of structural or supportive network changes [17,18]. However, there has been relatively little exploration of ways to improve the uptake and effectiveness of workplace interpersonal support programming for public safety personnel, despite implementation of many of these types of programs across Canada [6]. 

## 2. The Job Demand Control (Support) Model 

The Job Demand Control (JDC) model is an occupational health theory developed by Karasek [19]. The JDC developed from Selye’s [20] classic stress model, which argued that in response to an environmental stressor the body goes through a three-stage process (alarm, resistance, and exhaustion). While Selye’s model was developed for acute and potentially life-threatening stress reactions, Karasek’s JDC model was developed to understand and explain the effects of work environments where stressors are chronic, not life-threatening, and are often the product of organizational decisions that are alterable. The JDC theory posits that individuals devote cognitive resources to meet increasing demands, which results in an elevated level of physiological arousal and increased cardiovascular and nervous system attention. Over the long term, individuals run out of cognitive resources to meet these demands, resulting in poor physical and psychological health [19]. 

The JDC model is based on two psychosocial characteristics of work: decision latitude, or job control, (i.e., employees’ control over how and when they complete tasks) and job demands (i.e., workload as a psychological stressor). Job demands interact with decision latitude to create four distinct work conditions (job strain, active jobs, passive jobs, and low-strain jobs) that are protective or conducive to psychological stress [19]. The most psychologically harmful jobs are those characterized by job strain (i.e., when job demands are high and decision latitude is low); whereas active jobs (i.e., high job demands and high decision latitude) can result in positive psychological outcomes [19]. These work conditions reflect the idea that there is both “good” stress and “bad” stress [20]. In the mid-1980s, Johnson and Hall [21] enhanced the JDC model by including workplace support (i.e., helpful relationships at work on job-related matters, generally with supervisors and/or co-workers). Support was included as it can provide increased control through the collective control a social group may have over their environment that the individual may not have [22]. This expanded model also posited that jobs that are low in control, high in demand, and low in support (isostrain jobs) have the most harmful effects on employee health. This theory was named the Job Demand Control Support (JDCS) model [21]. 

The JDC and the JDCS each have two main hypotheses that can be empirically tested: (1) the (iso)strain hypothesis and (2) the buffering hypothesis [23]. Importantly, the two competing hypotheses have different implications for workplace design. The (iso)strain hypothesis posits that in conditions of (iso)strain, organizational interventions must increase job control (and social support, for the JDCS) while decreasing job demands to reduce the negative outcomes associated with the work conditions. In contrast, the buffering hypothesis posits that increasing control (or social support, for the JDCS) is sufficient to counteract the negative effects of job strain [23]. Decades of research have tested these competing hypotheses on stress-related illness risk across a wide range of occupational categories [23,24,25]. In general, there has been more consistent support for the job (iso)strain hypothesis although there has been partial support for the buffering hypothesis among specific occupational groups [23,24,25]. While there may be more consistent support for the (iso)strain hypothesis, this does not disprove the buffering hypothesis as they are not mutually exclusive, and the buffering hypothesis can be viewed as a specific form of the (iso)strain hypothesis [24]. We focus particular attention on the buffering hypothesis of the JDCS model as correctional institutions have implemented social support programs to decrease the psychological impacts of the correctional work environment. 

### 2.1. Relevance to Topic Area 

According to Karasek’s JDCS model, high job demands combine with low decision latitude and low social support to create work conditions that are conducive to job (iso)strain [19]. The job conditions experienced by correctional officers in Canada meet the requirements of job (iso)strain. Correctional institutes typically experience high operating costs, and many report budget cuts, overcrowding, and occupational shortages which increase the job demands of staff like correctional officers [26,27]. While officers are often left to work more with less, there have been simultaneous increases in formal procedures and bureaucratization of the system resulting in decreased decision latitude [28]. As a response to high demand and low job control with frequent exposure to violence, aggression, and traumatic experiences, increasing the social support offered to correctional officers has become one of the primary preventative methods used in an attempt to decrease the incidence of occupational stress injuries [13]. Workplace support interventions such as peer-support programs, critical incident debriefs, and mentorship training programs have been implemented to fill this need [13,14]. 

According to the buffering hypothesis of the JDCS model, social support is posited to be a moderator of the relationship between job demands and poor psychological well-being [21]. Specifically, social support is expected to be particularly helpful in ameliorating poor psychological well-being when job demands are high. The effect of support is hypothesized to be less important when job demands are low to moderate. Research testing the JDCS’ buffering hypothesis among correctional officers has found that social support can modify the relationship between job strain and psychological outcomes if the right type of support (i.e., functional support) [29] is provided by the right people (i.e., supervisory support) [30] in the right context (i.e., organizational climate conducive to engagement) [27]. A review of workplace mental health programs available to first responders (including correctional officers) highlights a disconnect between what is being offered and what is successful [13]. McCleary [13] found that most social support programs offered to correctional officers were provided by their peers. 

The limited studies available that evaluate the JDCS buffering hypothesis among correctional officers has not found evidence that co-worker support is successful in buffering job strain and improving psychological outcomes [30]. An environmental scan identified 18 social support programs offered to first responders (including firefighters, police and paramedics, but not correctional officers) in North America found that the social support interventions varied in scope and may have been implement by mental health professionals, trained providers, workplace peers or a combination of personnel [6]. Based on the current offerings of programs, the authors surveyed 134 workplace personnel with knowledge about peer support programs and found that 31% of them agreed that running the program resulted in an increased workload for peer supporters and that the stigma associated with mental health often prevented participation in the program [6]. A recent study that examined the availability and perceived use of mental health supports among PSP and found, although most PSP had access to professional, non-professional (e.g., Employee Family Assistance Programs), and peer support, many would only access professional or organizational support as a last resort, instead preferring to access support from a spouse or friend [14]. This suggests that PSP still perceived workplace stigma related to mental health and may be unwilling to seek support from peers and leaders even if that support is available.

In the context of correctional officers, co-worker support may not improve psychological well-being because it cannot address systemic issues related to high job demands and low job control. Supervisor support, particularly functional support, may be more successful [26,31,32] but there is often resistance to access such support even if it is available [14]. Research testing the (iso)strain hypothesis among correctional officers has found that high support had the greatest potential to reduce negative workplace outcomes (such as job changes [29], job satisfaction [30], and perceived danger [32]) when job demands are also reduced. Yet, conflicting results remain on the role of social support in alleviating negative psychological outcomes [29,30]. Based on this evidence of the buffering hypothesis in negative workplace outcomes, further research is needed to test the hypothesis that workplace mental health interventions for correctional officers may be more successful if they also focused on decreasing job demands rather than relying solely on increasing social support to buffer negative psychological outcomes. 

### 2.2. Strengths and Weaknesses of JDCS 

The JDCS is one of the most widely used theories of workplace health in public health and has had a significant impact on building empirical evidence of the connection between work stress and health [33]. The simplicity and broad applicability of the constructs that make up the JDCS theory make it a suitable choice for interdisciplinary research. The original JDC theory was developed in response to the absence in other occupational health theories of an emphasis on distinct components of job stress [33]. Indeed, the distinction between decision latitude and job demands is the first strength of this theory and years of research have shown the unique impact of each of these constructs on the psychological health of an employee [24]. The central constructs of this theory provide a way of measuring processes that are not entirely observable but act as intervening variables between the stimulus (i.e., workplace environment) and the response (i.e., psychological outcomes). Second, the JDCS has established, valid, and reliable scales that provide testable measures of the workplace environment. These scales have been tested across a wide range of occupations internationally and continue to show the predictive power of the constructs on psychological well-being [24,34,35]. The standardized scales allow for comparisons within occupations and across occupations. Finally, while debate remains on the support for the buffering hypothesis, the constructs can be applied to a variety of organizational interventions that can be implemented and evaluated for their effectiveness in improving psychological outcomes. 

A weakness of the JDCS theory is the vagueness of the constructs. Job demands, in particular, lacks a clear conceptual definition [36]. Notably, researchers have argued that the constructs of job demand and decision latitude may not be interpreted in the same way across organizational contexts [37,38]. Although the use of standardized measures is a strength in terms of generalizability, some researchers have argued the need to develop specific scales to assess diverse workplaces to better capture the nature of their work [30]. van der Doef and Maes [23] remarked that for jobs in human services, stressors related to interactions with clients could be an equally or more powerful job demand than those included in current measures. Second, while validated measures exist, researchers often use shorter versions of the measure or select some items from each construct to include while omitting others. This may explain some of the inconsistent findings in the literature [38]. 

While the JDCS provides an excellent frame for explaining the impact of workplace factors on individual health outcomes, the job stresses associated with correctional work do not exist in a vacuum. To develop mental health interventions that address the root of the stressors, there is a need to look at forces outside of the workplace setting to determine how these stressors developed and where public health can intervene. 

## 3. The Social Ecological Model

The Social Ecological Model (SEM) emphasizes the importance of environmental determinants on individual health behaviour [39]. Ecological models originated from the field of human ecology, which studies the relationship between humans and their environment [40]. While human ecology focuses on the biological environment, social ecology focuses on the influence of the social, cultural, and institutional environments on individuals [41]. 

McLeroy and colleagues [42] developed one of the most commonly used iterations of the SEM. The authors extended Bronfenbrenner’s system theory [43] into the field of health promotion to guide the development of behavioural interventions. They [42] highlighted five aspects of the SEM: (1) intrapersonal (i.e., individual knowledge, skills, and motivation), (2) interpersonal (i.e., relationships with others), (3) institutional (i.e., policies and procedures of organizations), (4) community (i.e., availability and location of resources within a defined boundary), and (5) policy (i.e., local, provincial or federal policies and laws). McLeroy and colleagues [42] propose the use of the SEM to help identify and guide the development of multilevel interventions where all levels are understood as determinants of health which can help or hinder individuals to modify their behaviours [44,45]. 

While SEMs provide researchers with a framework to consider multiple levels of influence on health behaviours and outcomes, SEMs are limited in not defining specific constructs or variables to generalize across multiple health behaviours or outcomes [39]. Instead, several principles can be applied to multiple health behaviours. First, factors at multiple levels influence health behaviour and the strength of this influence may vary by health behaviour and context. Second, social and physical environments are a significant determinant of individual behaviour and health outcomes. Hence, higher-order factors can help or hinder behaviour at lower levels of the SEM. Third, factors at multiple levels interact with one another but the exact nature or importance of those interactions may be difficult to determine. Fourth, SEMs should be tailored to the health behaviour or outcome, therefore, there is not one generalizable hypothesis across outcomes, and instead, it is unique to the behaviour or outcome under study. Despite this, a focus on multilevel interventions is hypothesized to be more effective, in terms of reach and sustainability, than single-level interventions [39]. 

### 3.1. Relevance to Topic Area 

There are few examples from the literature on the application of the SEM to high-stress and high-risk occupations, such as correctional work [46]. However, an SEM approach would benefit our understanding of how structural changes (i.e., forces outside of the control of the individual correctional officer) can influence the occupational environment, which, in turn, is co-constructed through the interactions between inmates and officers, and impacts mental health [47]. 

### 3.2. Policy 

The correctional system in Canada has undergone changes in the last 15 years which have had lasting impacts on the mental health and well-being of inmates and correctional officers. For example, in 2012, the Safe Streets and Communities Act (Bill C-10) imposed mandatory minimum sentences for certain drug-related offences, increased the length of sentences for drug trafficking offences and for sexually based offences, increased restrictions for applying for parole, and imposed harsher sentences on young offenders [48]. These policy changes increased the number of inmates in corrections [49] and the length of time inmates spent in remanded custody, and decreased the number of programs in place in correctional institutes for inmates with complex needs [50]. 

### 3.3. Community 

During the same period as the implementation of Bill C-10, the federal Deficit Reduction Action Plan (DRAP) included $295 million in cuts to the operating budget of Correctional Service Canada and many other social services [51]. These cuts lead to increases in the number of marginalized people (e.g., those with mental health conditions and/or drug or alcohol use problems), who might have been supported in the community, being institutionalized within the correctional system [48]. The implementation of Bill C-10 eroded social services and their ability to be socially supportive of the diverse needs of marginalized people, resulting in increased numbers of inmates in correctional institutions for longer periods with decreased funding for treatment.

### 3.4. Institutional 

The community conditions created by policy and budget changes have affected how often correctional officers are exposed to violence, how correctional officers respond to high-risk situations, the number of inmates with complex needs, and the effectiveness of the correctional system to rehabilitate the inmates housed within it. For example, Ricciardelli and colleagues [52] report that correctional budget cuts to rehabilitative programming in prisons has resulted in near-daily violent interactions between inmates and between inmates and officers. According to the Office of the Correctional Investigator’s Annual Report 2018–2019, use of force incidents in federal correctional facilities have increased by 91% in the past five years and incidents of self-injury and assaults by inmates have increased by 236% and 124%, respectively [53]. Frequent exposures to violence have produced new managerial directives (including administrative segregation) for situations where officers might have previously used verbal negotiation techniques [9]. This fundamentally changes how officers and inmates interact and has had significant and cyclical impacts on the mental health and well-being of officers and inmates. Nurse et al. [46] noted that, in light of increasing job demands and restricted job control, inmates spend more time in their cells with few supports to ease the boredom and frustration of their incarceration. Lambert et al. [32] found that officers who reported that their organizations had increasingly formalized procedures were more likely to report a heightened sense of workplace danger. Butler [54] surveyed a nationally representative sample of male offenders and found that confinement in restrictive housing (including administrative segregation) resulted in increases in assault misconduct. These conditions of confinement result in increased tension and potentially violent interactions, thus reinforcing the need for managerial directives, and thus reduced job control, in the first place. Moreover, hostile and restrictive conditions in correctional institutions increase recidivism and thus contribute to the problematic conditions of the correctional institutions [55].

### 3.5. Interpersonal

Correctional officers work in pairs and rely on their partners for functional support when dealing with potentially violent situations. Social capital must be maintained to ensure trust and cohesion among officers [56]. In its absence, the risk to officer safety is high. Therefore, social capital, which is a marker of micro intra-group relations, can be a powerful activator of behaviour. At times, adhering to occupational norms can even conflict with an individual officer’s private values and needs [57]. Occupational norms are shaped by the environment in which employees work and the interactions between actors in that environment. In prison, among officers and inmates, dominant masculine characteristics such as bravery, strength, aggression, and stoicism are highly valued [58,59]. Officer interactions are frequently contextualized by masculine stereotypes. In this sense, masculinities and femininities are not natural expressions of maleness and femaleness but rather social constructs achieved through job demands and interactions [60]. Traditionally masculine identities are sometimes adopted to avoid being viewed as vulnerable in the eyes of co-workers, inmates, and supervisors, and to maintain or build social capital [61]. 

Seeking social support for mental health problems may decrease an officer’s social capital and jeopardize their workplace persona [62,63]. PSP report substantial stigma in accessing support [64]. Moreover, correctional officers may view their co-workers unfavourably if they seek care for mental health conditions as it increases the job demands for the officers that remain if back-filling is not met [65]. This has been called the “circle of stress” (p. 480), where stressors such as reduced staffing, prison culture and management, and fear for safety result in low morale and staff shortages due to short-term or long-term disability, thus lowering job satisfaction and increasing the stress of remaining staff members who must cope with further increasing job demands [46]. 

### 3.6. Individual 

This section applied the SEM to correctional work to illustrate how structural changes impact interpersonal support program delivery and thus individual health outcomes. When examining the question of why workplace social support programs have not been effective at reducing the mental health outcomes experienced by correctional officers, the SEM provides a framework to look at this question across multiple levels of influence to see how factors, beyond the individual level, impact individual behaviour [17]. This deeper approach is needed to understand why merely increasing workplace social support may not buffer the mental health impacts of this occupation insofar as job demands remain high.

### 3.7. Strengths and Weaknesses of SEM 

A clear strength of the SEM is that it provides a framework for understanding the breadth of a health topic across multiple levels. It provides an alternative to the reliance on interpersonal or individual level interventions which have been criticized for victim-blaming due to their focus on changing individual behaviour in the absence of environmental alterations [42]. Although workplace social support programs for correctional officers are focused on the interpersonal level, the target of the interventions is still individual behaviour changes [42]. This can place an undue burden on correctional officers to manage their mental health in the face of unaddressed structural problems. Health groups and government agencies are increasingly relying on multilevel interventions to solve the most pressing health problems. The SEM is well suited for studies examining complex public health problems where individual or interpersonal level interventions have been unsuccessful. Multilevel interventions are posited as being more effective than interventions limited to a single level or factor [40]. Third, the SEM has been called a meta-model, in that it provides a framework for integrating other theories (e.g., the JDCS) and models that focus on psychological, social, or organizational levels of influence [44]. Therefore, in the context of workplace mental health programming, the SEM could be integrated with an organizational level theory which can provide more specificity regarding testable hypotheses and constructs. 

Despite these strengths, there are practical challenges of using the SEM. First, it lacks specificity of constructs and the hypothesized impact of these constructs at each level of influence. The main criticism of the SEM is that it is not a true theory. Rather, it is a model or framework that provides a broader perspective about a health issue. As a result, the SEM does not provide sufficient information to derive testable hypotheses and will benefit from the integration with a theory with validated measures and clear hypotheses. Second, one of the main tenets of the SEM posits that multilevel approaches have a larger effect than single-level approaches, remains untested due to the limited number of studies that have successfully implemented a multilevel intervention [44,66]. Finally, intervening across multiple levels of influence is not always feasible for a variety of reasons. To implement multilevel interventions, larger financial resources are required [67], which may be difficult to obtain given the limited information on the effectiveness of multilevel interventions [44]. Given the popularity of SEMs in public health, there may be political barriers in proposing interventions at structural levels. Public health professionals also may experience strict limits on their ability to intervene at the structural level (i.e., engage in policy advocacy) [44]. 

## 4. Discussion

This paper drew on two theories, the JDCS and SEM, to help understand intervention issues related to improving mental health among corrections workers in Canada. The SEM illustrated how structural policy changes can impact organizational policy, interpersonal norms, and individual actions, while the JDCS model illustrated that the occupational environment of correctional officers can be characterized as one of job (iso)strain which places correctional officers at elevated risk of mental health conditions. To alleviate conditions of job (iso)strain, current mental health programming has primarily focused on increasing individual coping and resiliency (i.e., an intervention at the individual level of the SEM) and increasing interpersonal support. However, both theories have advantages and disadvantages that suggest neither is likely to improve intervention efforts without greater specificity and a broader systems approach. 

Theorists have noted that the SEM pairs well with theories that focus on the target setting for the proposed intervention(s) [39]. The JDCS model explains the mechanism of how workplace factors result in job (iso)strain and individual psychological outcomes, which adds specificity to some levels of the SEM. However, to craft interventions or understand why current interpersonal interventions have failed, we must examine the structural drivers of individual behaviour and enhance the SEM with concepts that are more specific. 

Structural policy changes implemented in the past 15 years (e.g., changes in the criminal code, budget cuts, and decreases in community programming to support marginalized people) have had profound effects on the working conditions of correctional officers and decreased tangible resources to deal with an increasingly complex prison population [49,50]. Calls for re-allocation of policing budgets to mental health services and community support programs could have a positive mental health impact on correctional officers and inmates. Increasing community supports and treatment options for marginalized individuals could reduce job demands of correctional officers by reducing the number of individuals with complex needs housed in correctional institutions. Yet, addressing the growing mental health burden experienced by correctional officers, and PSP, in Canada cannot be successful if focused on a single level intervention. Moreover, efforts are needed to improve gendered occupational norms that prevent individuals from using interpersonal supports programs offered in the workplace. Implementing a multilevel approach by coupling an interpersonal stigma reduction with policy level changes to community mental health supports could decrease job demands and increase willingness to access social support, thus reducing adverse mental health outcomes among Canadian correctional officers. 

## 5. Conclusions

Together, the JDCS and SEM can contribute to the holistic understanding of interpersonal workplace mental health programming for correctional officers. Occupational theories such as the JDCS have great value for health promotion interventions, while taking a settings-based approach and applying the SEM is important for community and societal health promotion planning. Given the complexity of policies, practices, attitudes, and environments that can exert an impact on the functioning of correctional institutions, a combined approach can offer novel insights into how to develop, implement and evaluate new interventions aimed at improving the mental health of corrections workers. 
